# For Sake of Youth and for Sake of Policies and Programmes. Why Youth Participation is a Right, a Requirement and a Value: Comment on "Between Rhetoric and Reality: Learnings From Youth Participation in the Adolescent and Youth Health Policy in South Africa"

**DOI:** 10.34172/ijhpm.2023.7890

**Published:** 2023-05-21

**Authors:** Gabriele Prati, Cinzia Albanesi

**Affiliations:** Department of Psychology “Renzo Canestrari,” University of Bologna, Cesena (FC), Italy

**Keywords:** Youth Participation, Health Policy, Policy-Making Process, Actors, Youth, Empowerment

## Abstract

This commentary discusses an article by Jacobs and George which investigated how youth participation can be an important component of health policy-making by conducting a case study based on qualitative interviews. We appreciate the methodology and the main findings of the study, which contribute to advancing our understanding of the challenges and opportunities of youth participation in health policy-making. We note that this article raises several questions and issues that we must address to advance research and practice: (*i*) is there is a substantial gap between rhetoric and reality in terms of youth participation? (*ii*) do youth policies have a direct impact on youth participation? (*iii*) can we define and operationalise meaningful engagement? (*iv*) who is included and who is excluded in youth participation projects? and (*v*) is youth participation a right, a requirement and a value?

## Background

 Jacobs and George^1^ sought to investigate how youth participation can be included in health policy-making by examining youth participation in the Adolescent and Youth Health Policy (AYHP) formulation process. The authors collected and analysed 30 in-depth, semi-structured interviews with policy actors and highlighted the opportunities and challenges that arise from youth participation in the AYHP. The article reveals some important findings and advances our understanding of youth participation processes. In this commentary, we will focus on several aspects of research on youth participation in general and specifically with reference to this article.

 The authors argue that “There is a substantial gap between rhetoric and reality in terms of youth participation with scant research on youth participation in health policy-making, both globally and in South Africa.” The title itself stresses the tension between rhetoric and reality. However, we believe that such a gap between rhetoric and reality in terms of youth participation was not well highlighted and marked in the article. No evidence was provided for such rhetoric concerning youth participation by policy-makers, leading authorities, and politicians. This rhetoric was simply taken for granted. The rhetoric on youth participation is usually regarded in terms of the gap between an emphasis on youth participation and the practice. It would have been interesting to see evidence concerning policy-makers, leading authorities, and politicians’ rhetoric on youth participation.

 While the paper put the spotlight on the rhetoric on youth participation, it does not take into account the youth at risk rhetoric, which is probably prevalent. Bessant^2^ highlights the issue of risk discourses that draw attention to concerns about problem behaviours (ie, criminal youth, substance abuse, school dropout, unemployment) and as a result justify the absence of youth participation in policy development processes. In other words, the discourse of youth at risk legitimises current practices of denying youth their rights (having a voice, being heard and contributing to decisions that concern their life), while discourses on youth apathy and disengagement are perfect to blame youth and for their absence to justify it. Put in this way, there is little contrast between youth at risk rhetoric and a reality characterised by the absence or scarcity of opportunity for youth participation in policy development. Compared to the discourse of youth at risk, the rhetoric on youth participation even has the advantage of legitimising the view that youth participation is needed and relevant (and putting the responsibility on youth if they are not “there” when it is needed). Of course, we are not denying that a gap between the rhetoric on youth participation and the real praxis of youth policies may exist in specific situations.^3^ The point here is that such a gap has been assumed and not discussed and substantiated. If we want to stay on the level of rhetorical strategies, we need to acknowledge that the (even limited) rhetoric of relevance and need for youth participation blurs the focus on participation as a right. If participation is a right, as stated in the United Nations Convention on the Rights of the Child, it should be “granted” beside its relevance to reach out to any (kind of) young people, like for education where programmes and strategies exist, across the globe. Framing participation as a right would neutralise the rhetoric of youth at risk (no one could be excluded, including those at the margins) and substantiate discourses on social justice. Interestingly, the empirical data collected on the AYHP project revealed that participation is seen as instrumental to improving policy-making, without giving too much consideration about rights and justice, being limited to the scope of having a youth voice.^4^ Indeed, youth were consulted, and that was enough.

 The paper contributes to the discussion of how youth policies can enhance youth participation. It is important to problematise the assumption that youth policies have a direct impact on youth participation in terms of involvement in decision-making and representation of youth participation. Moreover, different patterns of youth participation may result from constellations of youth participation and policies as the expression “youth participation regimes” exemplifies.^5^ We agree in principle that participation should be meaningful because not all forms of participation can be regarded as such as a result of a variety of factors. However, youth participation can be defined differently according to various disciplines. In addition, youth participation can be regarded as a complex and multifaceted construct. For instance, different types of participatory activities (political, activist, political online and civic) among youth have been recognized. Including both manifest and latent engagement as well as formal and informal practices, such different participatory activities can result in different profiles of citizenship orientations (eg, active trustful, active distrustful, standby trustful, standby distrustful, unengaged trustful and unengaged distrustful).^6^ If youth participation is a very complex phenomenon, the concept of meaningful engagement is a fuzzy concept. Even if some significant efforts to clarify it exist, in particular with respect to youth political participation, we may speculate that the meaningfulness of youth engagement is context specific and should be defined by young people themselves. The institutional and hegemonic character of existing definitions of youth participation may ignore, devaluate, or even forbid many practices and expressions of youth.^5^ Therefore, the definition of meaningful engagement remains vague and ambiguous.

 The “ladder” metaphor^7^ for youth participation has proved to be useful in judging the levels of participation from manipulation through to young people-initiated and directed initiatives. Implicit in the ladder metaphor is that each rung is a progressive step towards meaningful participation. However, recent theorization and evidence regarding youth–adult participation research suggest that one participation type is not more ideal than another.^8^ For instance, the young people-initiated and directed initiatives at the top of the ladder proposed by Hart may be not ideal for empowerment. The type Pyramid, at least, differently from Hart’s ladder conceptualisation, does not assume what meaningful means, and youth-adults partnership is seen as meaningful in its capacity to empower young people. The way the contribution of Wang et al is presented is somehow misleading, because youth-adults partnership is not at the top of the pyramid. Indeed, this model aims to achieve a balance between youth and adult control, but because it is identified as the most promising for empowerment purposes. On the contrary, the youth-driven participation at the top of the ladder may be unrealistic and implausible in some situations. Therefore, a conceptualisation and a model of meaningful youth participation was much needed and should not be taken for granted.

 With regard to meaningful participation, the voices and perceptions of youth involved in the AYHP formulation process are important. We believe that future research should report data on youths’ voice and perceptions of their experience. Qualitative and quantitative methods can be used to document young people’s perspectives, perceived quality of participation, as well as other indicators of process and outcome.^9,10^ The sub-section that describes what worked well during the AYHP process does not report the voices and perspectives of young people. An AYHP Author Academic “described the process of participatory research as being mindful of the voice and agency of young people.” This is a very important point. But this is not enough. The voices and perspectives of young people are relevant here,^4^ still they represent a small group in the research sample, and one could question to what extent they may represent the diverse youth of South Africa.

 We agree with Jacobs and George^1^ that “AYHP was a step toward including youth in the development of health policy” but it is also clear from the analysis that this step was limited to an “elite” of young people, because AYHP did not take into account intersecting youth identities. In this sense, AYHP is aligned with most projects on youth involvement in deliberative and participatory processes that even when including young people, and it is not always the case, tend to miss an intersectional perspective. When analysing the process, many participants referred, more or less directly, to multifarious shortcomings of youth participation in the project: lack of diversity, and lack of consistent roles, also as a result of lack of capacity to stimulate the process of participation. Participants identified additional underlying reasons for shortcomings (eg, time pressure and the need to get the project done). However, the literature on youth participatory action research has consistently shown over time that the inclusion of young people in youth participatory action research requires adults to align with the youth agenda (their times, their tools, which of course may challenge the get it done priority), engage in trust building but also be prepared to manage conflict and dispute, as well as to questioning oppressive and exclusionary practices.^11^

 We also agree that intersectoral partnerships are relevant for the promotion of population health.^12^ The interviews with stakeholders clearly illuminate the challenges of collaboration and coordination across departments (“Sometimes it was like climbing Mount Kilimanjaro”). Although it seems a promising solution, the proposal for “a dedicated, capacitated, national coordinating mechanism department, ideally led by the Presidency” was not fleshed out. This would be an interesting avenue for future research. It would be interesting to investigate if and how such a coordinating mechanism department is effective in addressing the challenges of multi-sectoral coordination and collaboration. We may speculate that any coordinating mechanism that has the ambition to be effective should build on the existing literature that has identified the psychosocial, organisational, and economic conditions that are associated with effective intersectoral partnerships and coalitions.^13^ In addition, the contextual factors that may affect intersectoral partnerships should be considered. Last but not least, if young people are to be part of the coalition, the challenges to frame their participation as meaningful should be considered, starting probably from the analysis of failure.

 It would be useful for future studies to conduct a thorough analysis of youth participation initiatives through the lens of a theory of responsive or transformative participation.^14,15^ While adult-youth partnerships are deemed important for youth participation,^4^ adult people may hold negative views, attitudes, assumptions towards the expertise, knowledge, and capabilities of young people. Although youth people need to be considered experts (eg, of their own experiences), to ensure meaningful participation, ‘the professionals know best dilemma’^14^ can lead to implicit assumptions about who is and who is not the expert. In addition, adult people may feel they are losing control and power when promoting youth participation.^14^ Taken together, these feelings, assumptions, attitudes, and interaction patterns can lead to implicit practices regarding youth participation as responsive rather than transformative. Adult people ‘transform’ rather than ‘respond’ when the relationship is horizontal rather than vertical, when they meaningfully engage with young people rather than extract information from them, and when they trust youth rather than distrust them.

## Conclusions

 In their article, Jacobs and George^1^ investigated the phenomenon of youth participation by conducting a case study analysis of the AYHP. In the landscape of health policy in South Africa, AYHP represents an interesting and unique policy development process. The authors highlighted the positive features as well as the challenges involved in the process of youth participation in health policy-making. Research exploring if and how policy development processes such as the AYHP facilitate the leadership of young people in policies and promote meaningful participation is needed. Finally, although we agree that youth participation is a right, we believe that a challenge for future theory and research is to test and demonstrate the efficacy and effectiveness of youth participation in terms of youth empowerment and health as well as its benefits on the development, implementation, monitoring, and evaluating of policies and programmes.

## Ethical issues

 Not applicable.

## Competing interests

 Authors declare that they have no competing interests.

## Disclaimer

 The comments in this manuscript reflect only the personal position of the authors and do not represent the views of the university.
